# Bovine PMN responses to extracellular vesicles released by *Besnoitia besnoiti* tachyzoites and *B. besnoiti*-infected host cells

**DOI:** 10.3389/fimmu.2024.1509355

**Published:** 2024-12-19

**Authors:** Gabriel Espinosa, Constanza Salinas-Varas, Lisbeth Rojas-Barón, Christian Preußer, Elke Pogge von Strandmann, Ulrich Gärtner, Iván Conejeros, Carlos Hermosilla, Anja Taubert

**Affiliations:** ^1^ Institute of Parasitology, Justus Liebig University Giessen, Giessen, Germany; ^2^ Core Facility Extracellular Vesicles, Center for Tumor Biology and Immunology, Philipps University of Marburg, Marburg, Germany; ^3^ Institute of Anatomy and Cell Biology, Justus Liebig University Giessen, Giessen, Germany

**Keywords:** PMN, *Besnoitia besnoiti*, endothelial cells, extracellular vesicles, NET formation

## Abstract

Bovine besnoitiosis is a re-emerging cattle disease caused by the apicomplexan parasite *Besnoitia besnoiti*, which severely affects individual animal welfare and profitability in cattle industry. We recently showed that *B. besnoiti* tachyzoite exposure to bovine polymorphonuclear neutrophils (PMN) effectively triggers neutrophil extracellular trap (NET) formation, leading to parasite immobilization hampering host cell infection. So far, the triggers of this defense mechanism remain unclear. Emerging evidence indicates that extracellular vesicles (EVs) modulate PMN effector functions, such as ROS production or NET formation. Therefore, we tested whether exposure of bovine PMN to EVs from different cellular sources affects classical PMN effector functions and cytokine/chemokine secretion. EVs were isolated from *B. besnoiti*-infected and non-infected host cells (bovine umbilical vein endothelial cells, BUVEC), from tachyzoite-exposed bovine PMN and from *B. besnoiti* tachyzoites. EV concentration and size was determined by Nano-Flow cytometry and EV nature was confirmed by both classical EV markers (CD9 and CD81) and transmission electron microscopy (TEM). Overall, PMN stimulation with both BUVEC- and tachyzoite-derived EVs significantly induced extracellular DNA release while EVs from PMN failed to affect NET formation. BUVEC and tachyzoite EV-driven NET formation was confirmed microscopically by the presence of DNA decorated with neutrophil elastase (NE) and histones in typical NET structures. Moreover, confocal microscopy revealed EVs to be internalized by bovine PMN. Referring to PMN activation, EVs from the different cellular sources all failed to affect glycolytic or oxidative responses of bovine PMN as detected by Seahorse^®^-based analytics and luminol-based chemoluminescence, thereby denying any role of NADPH oxidase (NOX) activity in EV-driven NET formation. Finally, exposure to *B. besnoiti*-infected BUVEC-derived EVs induced IL-1β and IL-6 release, but failed to drive CXCL8 release of bovine PMN. Hence, we overall demonstrated that EVs of selected cellular origin owned the capacity to trigger NOX-independent NET formation, were incorporated by PMN and selectively fostered IL-1β and IL-6 release.

## 1 Introduction

*Besnoitia besnoiti* is an obligate intracellular parasite, closely related to *Neospora caninum* and *Toxoplasma gondii (T. gondii)*, and represents the etiological agent of besnoitiosis (1). Bovine besnoitiosis is a severe but mostly non-fatal disease of cattle, which is widespread in Africa, Asia and Europe (2). Despite a moderate mortality (< 10%), the high morbidity of this disease in cattle herds led to its classification as emerging disease by the European Food Safety Authority (EFSA) (3). *B. besnoiti* is transmitted by bites of tabanids (*Tabanus* spp.) or muscids, such as the stable fly (*Stomoxys calcitrans*) (4); sexual transmission via mating is still under debate. Clinical signs include acute non-specific symptoms (e. g. hyperthermia, weight loss, depression, anasarca) and chronic stages being characterized by severe skin alterations including alopecia, inflammation, hyperkeratosis and progressive skin thickening (5, 6). Moreover, reproductive issues, such as bull infertility or abortion and impaired milk production represent major characteristics of the disease (7). Consequently, besnoitiosis compromises individual animal welfare and causes significant economic losses in cattle industry (2).

The innate immune system plays a pivotal role in early elimination of parasitic infections (8). Polymorphonuclear neutrophils (PMN) represent the most abundant leukocyte type in the blood of most mammals (9) and are among the first immune cells to arrive at sites of infection. Besides classical PMN defense mechanisms like immunomodulatory molecule release (e. g. cytokines and chemokines), phagocytosis and reactive oxygen species (ROS) production, PMN release neutrophil extracellular traps (NETs) (10–12). NETs are web-like structures composed by DNA, histones and microbicidal peptides, which immobilize and eventually kill pathogens, thereby limiting their spread in infected hosts (13). The process of NET formation is generally described as NADPH oxidase (NOX)-dependent, however, some reports also documented NOX-independent NET formation (14–16). During classical suicidal NET formation, nuclear chromatin decondensation is induced, which is followed by neutrophil elastase (NE) and myeloperoxidase (MPO) translocation into the nucleus to fuse with chromatin before being released. Ultimately, PMN membrane disintegration can be mediated either by elevated ROS production or by the activity of lytic proteins like gasdermin, which induce membrane pores and subsequently result in NET extrusion into the extracellular matrix (17–21). Meanwhile, several protozoan parasites were reported to stimulate NET release (22–27), including *B. besnoiti* stages (28–31). *B. besnoiti* triggers NET release in a stage-independent manner, since tachyzoites and bradyzoites were equally proven to drive NET formation in bovine PMN (32). To date, the precise triggers of *B. besnoiti*-mediated NET formation remain to be elucidated. In this context, diverse biomolecules being released from or expressed on the surface of parasites or host cells (in case of *B. besnoiti* cattle infection: mainly endothelial cells, ECs) play a pivotal role in mediating host-parasite interactions. Upon stimulation, ECs are able to produce and secrete a broad spectrum of cytokines, chemokines and adhesion molecules (e. g. ICAM-1, E-selectin, Interleukins), which regulate inflammation and the recruitment of immune cells (33–35) like circulating PMN to affected areas (36). Consequently, the continuous cross-talk between immune cells and ECs is a key factor for the maintenance of tissue homeostasis (37). Furthermore, a broad range of different organism and cells, including parasites, ECs and PMN, are able to release extracellular vesicles (EVs) for communication purposes (38–40). EVs are nano-scaled membraneous vesicles containing a complex mixture of DNA, RNA, lipids, metabolites and proteins, with pivotal importance in cell-to-cell communication (41). Hence, it was reported that EV-driven reciprocal communication between PMN and ECs stimulates the extravasation of PMN to sites of infection (40). Moreover, emerging evidence indicates that EVs also modulate PMN effector functions, such as NET formation (42). Accordingly, parasite-derived EVs are able to transfer virulence factors, drug-resistance genes and differentiation factors between parasites, besides modulating host immune responses by stimulating the release of anti-inflammatory cytokines, which may then assist parasites in evading the immune system (38, 43). However, current information on the precise role of EVs in parasite-host communication is still limited. In this scenario, EVs from ECs (host cells) and PMN may represent key innate immune components playing a pivotal role during early stages of parasitic infection. Therefore, the aim of the present study was to determine effects of EVs of differential cell origin on both, host cell and PMN functions.

## 2 Material and methods

### 2.1 Ethics statement

This study was performed in accordance to the Justus Liebig University Giessen Animal Care Committee Guidelines. Protocols were approved by the Ethic Commission for Experimental Animal Studies of the Federal State of Hesse (Regierungspräsidium Giessen; GI 18/10 Nr. V 2/2022; JLU-No. 0002_V) and are in accordance to European Animal Welfare Legislation: ART13TFEU and currently applicable German Animal Protection Laws.

### 2.2 Primary bovine umbilical vein endothelial cell isolation and maintenance

Primary bovine umbilical vein endothelial cells (BUVEC) were isolated from umbilical veins obtained from calves born by *sectio caesarea* at the Justus Liebig University Giessen. Therefore, umbilical cords were stored at 4°C in 0.9% HBSS–HEPES buffer (pH 7.4; Gibco, Grand Island, NY, USA) supplemented with 1% penicillin (500 U/ml; Sigma, St. Louis, MO, USA) and streptomycin (500 μg/ml; Sigma) for a maximum of 16  h before use. For endothelial cells isolation, 0.025% collagenase type II (Worthington Biochemical Corporation) suspended in Puck´s solution (Gibco) was infused into the lumen of ligated umbilical veins and incubated for 20  min at 37°C/5% CO_2_ atmosphere. Cells were collected in cell culture medium supplemented with 1 ml fetal calf serum (FCS, N4637 Sigma) to inactivate collagenase. After two washes (350 × g, 12  min, 20°C), cells were re-suspended in complete endothelial cell growth medium (C-22010, ECGM, PromoCell) supplemented with 10% FCS. Then, cells were plated in 25  cm^2^ tissue plastic culture flasks (Greiner) and cultured at 37°C/5% CO_2_ atmosphere in modified ECGM medium (diluted at 30% in M199 medium) supplemented with 5% fetal bovine serum (FBS, 10270-106, Gibco) and 1% penicillin/streptomycin. Medium was replaced every 2–3 days. BUVEC cell layers were used for infection after 3 passages *in vitro*.

### 2.3 *Besnoitia besnoiti* tachyzoite maintenance

All experiments of the current study were performed with tachyzoite stages of the apicomplexan parasite *B. besnoiti* (Evora04 strain). Madin-Darby bovine kidney (MDBK) cells were used as host cells for tachyzoite *in vitro* production. Host cells were cultured in 75 cm^2^ plastic tissue culture flasks (Greiner) at 37°C/5% CO_2_ atmosphere in RPMI-1640 (R0883, Sigma-Aldrich) medium supplemented with 5% FBS and 1% penicillin/streptomycin. MDBK cell layers were infected at 80% confluency with 2.4×10^7^ tachyzoites. Parasites released from MDBK cells were scrapped and harvested from cell supernatants, filtered by a 5 μm syringe filter (Merck Millipore), washed, and pelleted (400×g, 12 min) prior to re-suspension in the working medium required. Tachyzoite numbers were determined in a Neubauer chamber, and parasite stages were placed at 37°C/5% CO_2_ atmosphere for further experimental use.

### 2.4 Bovine PMN isolation

Healthy adult dairy cows served as blood donors. Animals were bled by puncture of the jugular vein and peripheral blood was collected in heparinized sterile plastic tubes (Kabe Labortechnik). Heparinized blood was re-suspended at 1:1 ratio in 20 ml sterile PBS with 0.02% EDTA (CarlRoth), carefully layered on top of 12 ml Histopaque-1077 separating solution (density = 1.077 g/l; 10771, Sigma-Aldrich) and centrifuged (800×g, 45 min) without brake. After removal of plasma and peripheral blood mononuclear cells, the volume of the cell suspension was adjusted to 10 ml with Hank’s balanced salt solution (HBSS, 14065-049, Gibco). Then, 20 ml of lysis buffer (5.5 mM NaH_2_PO_4_, 10.8 mM KH_2_PO_4_, pH 7.2) were added and the sample was gently mixed for 60 s to lyse erythrocytes. Osmolarity was rapidly restored by addition of 10 ml hypertonic buffer (462 mM NaCl, 5.5 mM NaH_2_PO_4_, 10.8 mM KH_2_PO_4_, pH 7.2) and 10 ml HBSS. The lysis step was repeated twice until no erythrocytes were visible. PMN were then suspended in 5 ml HBSS, counted in a Neubauer chamber and allowed to rest on ice for 30 min prior to any experimental use.

### 2.5 Extracellular vesicle (EV) isolation

To isolate EVs from *B. besnoiti*-infected BUVEC, 8 x 10^6^ BUVEC (*n* = 3) in 75 cm^2^ plastic tissue culture flasks were infected with tachyzoites (ratio: 1:6) in modified ECGM medium (C-22210, Promocell) for 4 h (37°C, 5% CO_2_ atmosphere). After washing with sterile PBS, cells were resuspended in vesicle-depleted modified ECGM medium (EV medium) and incubated for 24 h. Equal numbers of plain tachyzoites and non-infected BUVEC were equally treated for controls. For isolation of EVs from tachyzoite-exposed PMN (*n* = 3), 5 x 10^7^ PMN were incubated with tachyzoites (ratio 1:6) in EV medium for 4 h. Equal numbers of plain PMN and of zymosan-stimulated PMN (0.1 mg/ml, 4 h) were used as controls. After incubation, EV-enriched supernatants were collected and pooled by experimental condition in conical tubes and differentially centrifuged (300×g for 5 min, 2,000×g for 10 min and 10,000×g for 30 min) to eliminate cell debris. Supernatants were concentrated using Amicon Ultra-15 100 kDa MWCO filter devices (Millipore, Billerica, MA, USA) to a final volume of 500 µl. Then, EV isolation was performed by size-exclusion chromatography (SEC) with an Automatic Fraction Collector V2 (Izon) using a qEV sepharose column (ICO-70, qEVoriginal/70 nm, Izon) according to IZON´s protocol. The columns were equilibrated with filtered (0.22 µm) PBS, pH 7.4 before loading EV samples (500 µl). After discarding 2.9 ml eluate, 0.5 ml fractions were collected into 2 ml Eppendorf tubes. EV fractions were concentrated using Amicon Ultra-15 100 kDa MWCO filter devices to a final volume of 100-150 µl. EV samples were stored at −20°C until further use.

### 2.6 Nano-flow cytometry

For EV characterization, Nano-flow cytometry was conducted using a Flow NanoAnalyzer (NanoFCM Co., Ltd, Nottingham, UK) equipped with a 488 nm and a 638 nm laser. The instrument was calibrated using 200 nm polystyrene beads (NanoFCM Co. Ltd.) at a defined concentration of 2.08 x 10^8^ particles/ml, serving as reference for particle concentration. Monodispersed silica beads (NanoFCM Co., Ltd) of four different diameters (68 nm, 91 nm, 113 nm and 155 nm) were utilized as size reference standards. Measurements of freshly filtered (0.1 μm), plain 1x TE buffer pH 7.4 (Lonza, Basel, Switzerland) were defined as background signals; consequently, respective values were subtracted from all other measurements. Particle concentration and size distribution of EV samples (diluted in 0.1 μm pore size-filtered 1x TE buffer) were calculated using NanoFCM software (NF Profession V2.0), based on data collected for one minute under a sample pressure of 1.0 kPa.

### 2.7 NET detection by immunofluorescence microscopy

Bovine PMN (*n* = 3) were co-cultured with BUVEC- and *B. besnoiti* tachyzoite-derived EVs (1 x 10^8^) in RPMI-1640 medium (without phenol red, R7509, Sigma-Aldrich) for 4 h (37°C, 5% CO_2_ atmosphere) on poly-_L_-lysine (0.01%) -pretreated coverslips (15 mm diameter, Thermo Fisher Scientific), fixed in 4% paraformaldehyde (Merck) and stored at 4°C until further use. For NET visualization, DAPI (Fluoromount G, ThermoFisher, 495952) was applied to stain DNA; anti-histone (clone H11-4, 1:100, Merck Millipore MAB3422, Darmstadt, Germany) and anti-NE (ab68672, 1:200, Abcam, Cambridge, UK) primary antibodies were used to detect respective proteins on NET structures. Therefore, fixed samples were washed three times with PBS, blocked with 1% bovine serum albumin (BSA, Sigma-Aldrich, Steinheim, Germany, 30 min, RT), and incubated in corresponding primary antibody solutions (1 h, RT). After three washings in PBS, samples were reacted with secondary antibody solutions (Alexa Fluor 488 goat anti-rabbit IgG and Alexa Fluor 594 goat anti-mouse IgG, Life Technologies, Eugene, USA; 60 min, 1:500, RT). Finally, samples were washed three times in PBS and mounted in DAPI-containing mounting media (Fluoromount G, ThermoFisher, 495952). Image acquisition was achieved by a BZ-X800 microscope (Keyence), thereby applying identical brightness and contrast conditions within the datasets of each biological experiment. Percentages of NET-forming PMN were calculated semi-automatically by dividing the events counted in the histone channel (multiplied by 100) by the events counted in the DAPI channel (44).

### 2.8 Extracellular DNA quantification

Bovine PMN (*n* = 3) suspended in RPMI-1640 were confronted with 1 x 10^8^ EVs from all cellular sources (see 2.5) and incubated for 4 h (37°C, 5% CO_2_). After incubation, picogreen (Invitrogen, Eugene, USA, 1:200 dilution in 10 mM Tris base buffered with 1 mM EDTA, 100 μl/well) was added to each sample. Extracellular DNA was quantified by picogreen-derived fluorescence intensities using an automated multiplate reader (Varioskan, Thermo Scientific) at 484 nm excitation/520 nm emission as described elsewhere (24, 28, 45).

### 2.9 Quantification of PMN oxygen consumption rates (OCR) and extracellular acidification rates (ECAR)

Oxidative and glycolytic responses of bovine PMN were monitored using a Seahorse XFp analyzer (Agilent). Briefly, 1 x 10^6^ PMN from three blood donors were pelleted (500 × g, 10 min) and re-suspended in 0.25 ml of XF assay medium (Agilent) supplemented with 2 mM of _L_-glutamine, 1 mM pyruvate and 10 mM glucose. 2 x 10^5^ cells were gently placed in each well of an eight-well XF analyzer plate (Agilent) pre-coated for 30 min with 0.001% poly-_L_-lysine (Sigma-Aldrich). Then, XF assay medium (Agilent) was adjusted to 180 μl total volume per well and cells were incubated at 37°C without CO_2_ supplementation for 45 min before Seahorse measurements. 1 x 10^8^ EVs from different cellular sources (see 2.5) were supplemented to the cells via instrument-own injection ports following 4 baseline measurements. The total assay duration was 160 min. Background subtraction and determination of OCR/ECAR registries were performed by using Seahorse Agilent analytics platform (https://seahorseanalytics.agilent.com).

### 2.10 Immunoblotting

The protein concentration of each EV isolate was estimated by the absorbance at 562 nm using the micro BCA protein assay kit (ref 23235, Thermo Scientific) following manufacturer’s protocol. EV-derived protein samples were supplemented with Laemmli-β-mercaptoethanol loading buffer (1x final concentration, #1610747, BioRad). Commercially available human EV-derived proteins (EV pos, EXOAB-POS-1, System Biosciences) and *B. besnoiti* tachyzoite protein extracts were used as positive controls. After boiling (95°C) for 5 min, proteins (20 µg/slot) were separated in 4-20% polyacrylamide gels (#4561095, BioRad) via electrophoresis (120 V constant for 1 h, tetra system BioRad) and then transferred to 0.2 µm PVDF membranes (trans-blot turbo #1704156, 2.5 A constant, up to 25 V, 7 min, BioRad). Samples were blocked in 3% BSA in TBS [50 mM Tris-Cl, pH 7.6; 150 mM NaCl containing 0.1% Tween (blocking solution); Sigma-Aldrich] for 1 h at RT, and then reacted with primary antibodies diluted in blocking solution (overnight, 4°C). Primary antibodies were anti-CD9 (ThermoFisher, Cat #MA1-19301, Mouse, 1:500), anti-CD81 (ThermoFisher, Cat #MA5-28419, Rabbit, 1:500), and anti-vinculin (Santa Cruz, Cat #sc-73614, Mouse, 1:500). Vinculin was detected as sample loading control. Following three washes in TBS-Tween 0.1% buffer, blots were incubated with secondary antibody solutions (dilution in blocking solution, 30 min, RT). Secondary antibodies were anti-Mouse (Pierce, Cat #31430, 1:40000) and anti-Rabbit (Pierce, Cat #31466, 1:40000). After three further washes in TBS-Tween (0.1%) buffer, signal detection was accomplished by an enhanced chemiluminescence detection system (Clarity Max Western ECL substrate, #1705062, BioRad) and recorded using a ChemiDOC Imager (BioRad). Protein masses were controlled by a protein ladder (PageRuler Plus Prestained Protein Ladder ~10-180 kDa, #26616, Thermo Fisher Scientific).

### 2.11 Quantification of ROS production

Total ROS measurement was performed by a chemiluminescence-based assay using luminol (A4685, Sigma-Aldrich). Therefore, 1 x 10^7^ PMN were suspended in 1 ml of HBSS; 100 µl (1 × 10^6^ PMN) was transferred per well to a white 96 well plate. Then, 90 µl luminol (80 µM final concentration) were added per well. For negative controls, non-stimulated PMN were used. After 30 readings accounting for 12 min, 10 µl of 1 x 10^8^ EVs isolated from BUVEC controls, *B. besnoiti*-infected BUVEC, *B. besnoiti* tachyzoites or Zymosan (0.1 mg/ml, Z4250, Sigma) were added to PMN. Chemiluminescence was measured for 3 h in a luminometer (Luminoskan, Thermo Scientific).

### 2.12 Transmission electron microscopy (TEM)

TEM analysis was performed on 1 x 10^10^ EVs derived from infected BUVEC and *B. besnoiti* tachyzoites. EV samples (10 μl) were fixed in a drop of 0.1 M cacodylate buffer containing 4% formaldehyde and 1.5% glutaraldehyde. Specimen suspensions were absorbed immediately after fixation on formvar-coated grids and stained with 1% ammonium molybdate. Negatively stained samples were inspected in a transmission electron microscope (EM 902N, Zeiss, Oberkochen, Germany) equipped with a slow-scan 2K CCD camera (TRS, Tröndle, Moorenweis, Germany).

### 2.13 EV labeling with far red dye

1 x 10^9^ EVs isolated from non-infected BUVEC, *B. besnoiti*-infected BUVEC and *B. besnoiti* tachyzoites were stained by far red staining (CellTrace™ far red, C34564, ThermoFisher) as described before (46). Briefly, EVs (15 µl) in PBS were mixed with 15 μl far red dye solution (40 μM) and incubated for 2 h at 37°C. To remove unbound dye, EV samples were loaded on a qEV sepharose column (qEVoriginal/70 nm, Izon) and processed as described before (see 2.5). Cell Trace far red dye alone was used as dye control.

### 2.14 Analysis of PMN EV uptake

1 x 10^6^ PMN were incubated with 1 x 10^9^ far red-labeled EVs derived from non-infected BUVEC, *B. besnoiti*-infected BUVEC and *B. besnoiti* tachyzoites in RPMI-1640 medium (without phenol red, R7509, Sigma-Aldrich) at 37°C for 6 h. Far red dye alone was used as dye control and unstimulated/unstained PMN were used as negative controls. Samples were mixed by gently pipetting every hour. Cells were washed twice with PBS before analysis. Confocal microscopy was performed with 0.5 x 10^6^ EV-stimulated PMN seeded in 24-well plates containing poly-_L_-lysine (0.01%, 20 min, RT and subsequent washing with PBS) -pretreated 15 mm coverslip. After 30 min of incubation, cells were fixed in 4% paraformaldehyde (Merck) and mounted in anti-fading buffer with DAPI (Fluoromount G, ThermoFisher, 495952). Images were acquired by a Nikon Eclipse Ti2-A inverted microscope equipped with ReScan confocal microscopic instrumentation (RCM 1.1 Visible, Confocal.nl) and a motorized z-stage (DI1500). Two channels were recorded for signal detection: DAPI/405 nm and far red dye/640 nm. Images were acquired by a sCMOS camera (PCO edge) using a CFI Plan Apochromat X20 and x60 lambda-immersion oil objective (NA 1.4/0.13; Nikon) controlled by NIS-Elements v 5.11 (Nikon, Tokyo, Japan) software. Identical brightness and contrast conditions were applied for each data set within one experiment using Fiji software.

### 2.15 IL-1β, IL-6 and CXCL8 measurements in cell supernatants

1 x 10^6^ PMN or BUVEC plated in 12 well plates (at 80% confluence) were exposed to 1 x 10^8^ EVs derived from non-infected BUVEC, *B. besnoiti*-infected BUVEC and *B. besnoiti* tachyzoites for 4 h (PMN) and 24 h (BUVEC) at 37°C, 5% CO_2_. LPS (1 µg/ml) and PMA/ionomycin (100 nM/5 µM) for PMN and LPS (0.01 µg/ml) for BUVEC were use as positive controls. After incubation, aliquots (100 µl) of cell supernatants (each *n* = 3) were analyzed for the presence of interleukin (IL)-1β (bovine IL-1β ELISA Kit, #ESS0027, Thermo Fisher Scientific, MA, USA), IL-6 (#ESS0029, Thermo Fisher Scientific, MA, USA) and CXCL8 (IL-8, bovine IL-8 ELISA kit, ABIN6957183, Antibodies Online, Germany), following manufacturer’s instructions. In the case of IL-1β and IL-6 ELISA kit, a high binding 96 well plate (#655061, Greiner bio one) were used. The samples were analyzed at 450 nm and 550 nm in an automatic Varioskan Flash Reader (Thermo Fisher Scientific, MA, USA). Standard curves and sample concentrations of IL-1β, IL-6 and CXCL8 were calculated using GraphPad PRISM^®^ V10.3.0 software package (GraphPad software, USA).

### 2.16 Statistical analysis

All experiments were repeated at least three times. Statistical significance was defined by a *p* value ≤ 0.05. The *p* values were determined by one-way ANOVA followed by a Dunnett´s multiple comparison test, with single pooled variance. Bars graphs represent the mean ± SD, and statistical analyses were generated using GraphPad PRISM^®^ V10.3.0.

## 3 Results

### 3.1 Isolation and characterization of EVs

**Figure 1:**
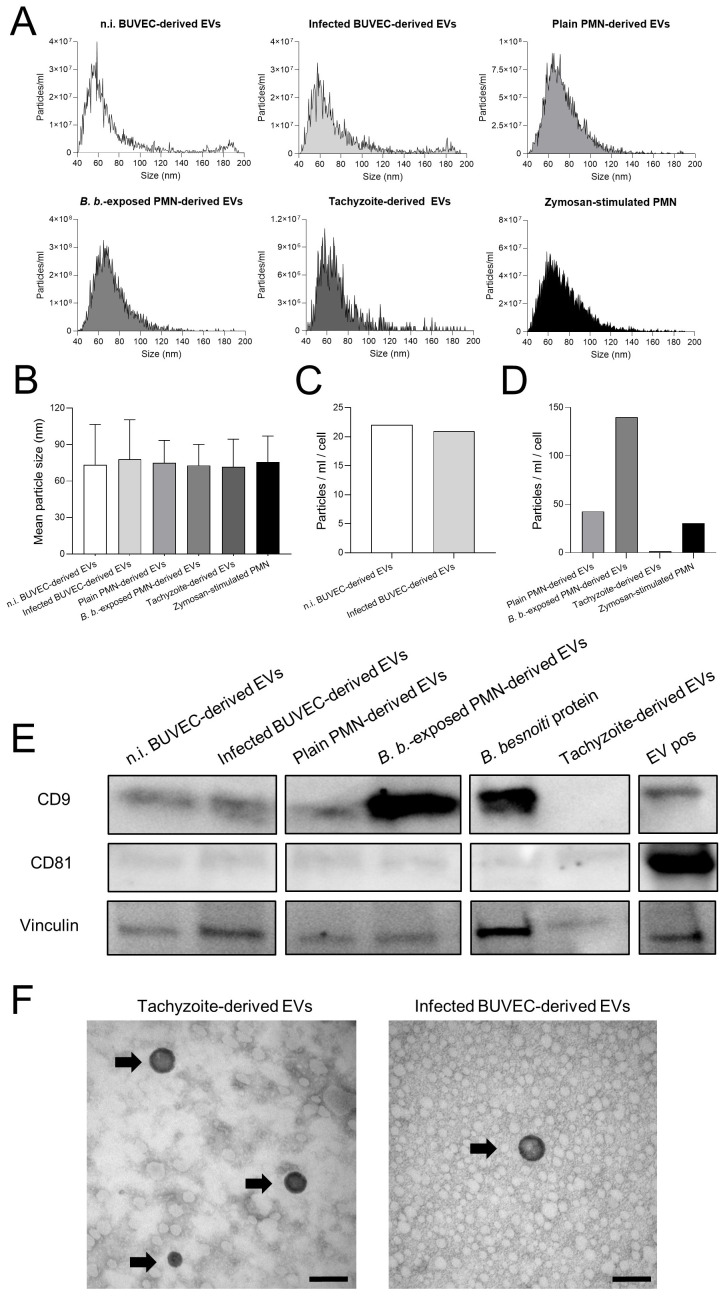
Characterization of BUVEC-, PMN- and *B. besnoiti* tachyzoite-derived EVs. Extracellular vesicles were isolated from non-infected BUVEC (n.i. BUVEC), *B. besnoiti*-infected BUVEC (Infected BUVEC), non-exposed PMN (Plain PMN), *B. besnoiti* tachyzoite-exposed PMN (*B. b*.-exposed PMN) and from plain *B. besnoiti* tachyzoites (Tachyzoite). **(A)** Exemplary histograms on EV size distribution, **(B)** particle concentration and **(C, D)** particle release per cell as assessed by Nano-Flow cytometry. Zymosan-stimulated PMN served as positive control for PMN-derived EV production. Mean particle diameters of EVs showed values around 70 nm. **(E)** Western blot analysis of BUVEC-, PMN- and *B. besnoiti* tachyzoite-derived EV samples probed with anti-CD9, anti-CD81 and anti-vinculin antibodies. Commercially available human EV-derived proteins (EV pos) and *B. besnoiti* protein extract were used as controls. **(F)** *B. besnoiti* tachyzoite-derived and infected BUVEC-derived EVs were studied by TEM (black arrows), and showed a typical EV morphology (scale bars indicate 100 nm).

EVs were isolated from all cell sources using size exclusion chromatography (SEC) with qEV/70 nm Original (IZON) columns, which are optimized for high EV recovery and minimal lipoprotein contamination. EV numbers and sizes from pooled fractions were assessed by Nano-Flow cytometry, EV-specific marker detection was performed by western blotting and EV morphology was visualized by TEM (**Figure 1**). In all cases, EVs peaked in size around 60-80 nm (**Figure 1A**). Overall, mean EV sizes from control BUVEC, *B. besnoiti-*infected BUVEC, plain PMN, *B. besnoiti*-confronted PMN, zymosan-stimulated PMN and *B. besnoiti* tachyzoites were detected, consistent with literature data describing a general size of 30-120 nm for small EVs (**Figure 1B**) (47). The mean concentration of particles per cell after 24 h incubation showed comparable EV secretion from BUVEC regardless of infection (**Figure 1C**). In contrast, EV secretion experienced a 3-fold increment when PMN were exposed for 4 h to *B. besnoiti* tachyzoites in comparison to plain PMN or zymosan-stimulated PMN (**Figure 1D**). Western blot analyses confirmed the EV nature of the particles since the samples proved positive for CD9 and showed weak signals for CD81 (besides vinculin as loading control), both representing typical EV markers (**Figure 1E**). TEM analyses revealed for the first time *B. besnoiti* tachyzoite-derived and *B. besnoiti*-infected BUVEC-derived EVs by confirming the typical EV morphology (**Figure 1F**).

### 3.2 Exposure of PMN to EVs from different cellular sources does not affect PMN oxidative and glycolytic responses

**Figure 2:**
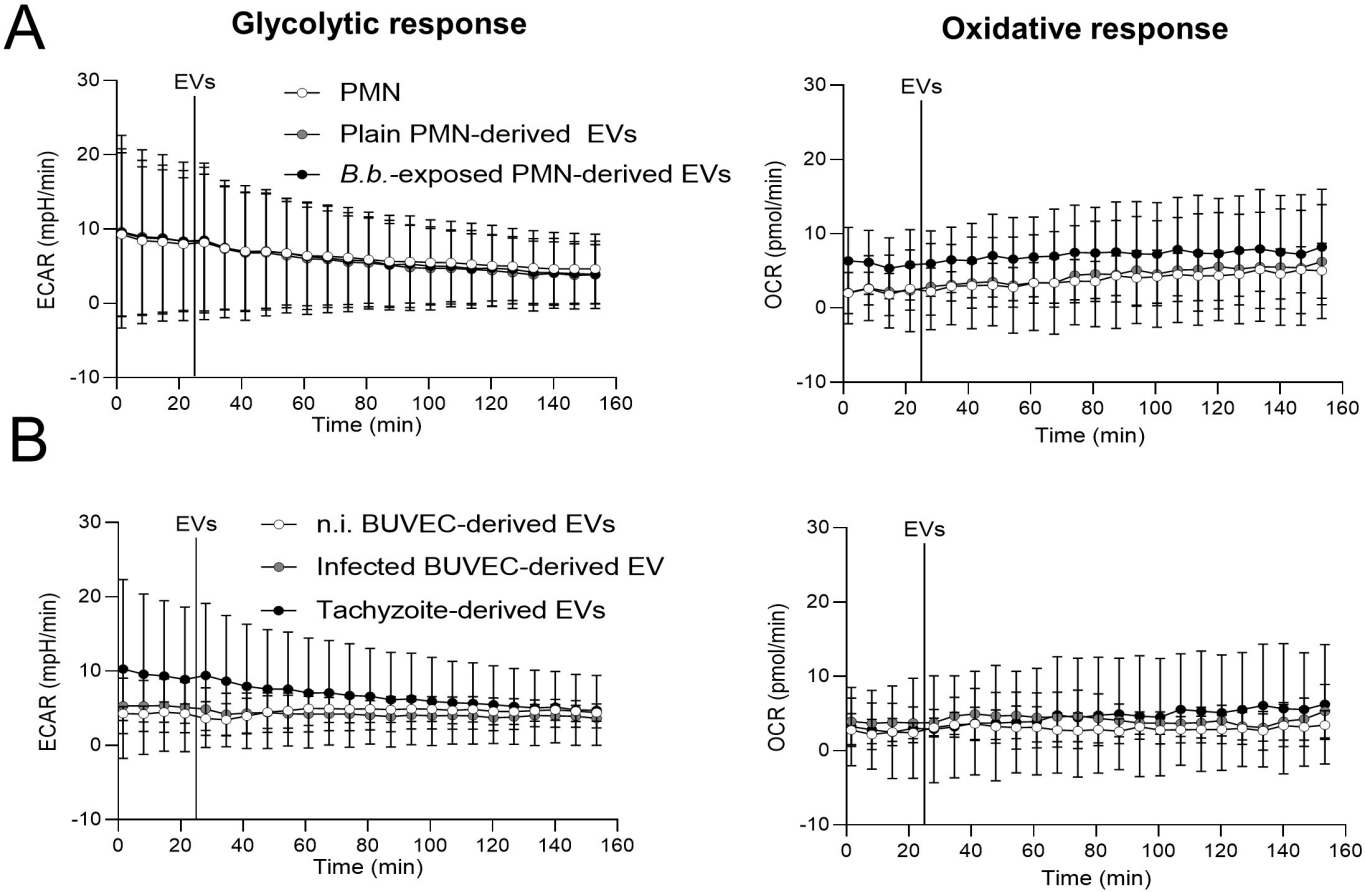
Exposure to EVs does not affect oxidative and glycolytic responses in bovine PMN. In absence of CO_2_, PMN were incubated in XF RPMI media for 45 min. Four basal measurements were performed and then PMN-derived EVs **(A)** or BUVEC-derived EVs **(B)** were supplemented to bovine PMN at the time point indicated by a vertical line. OCR and ECAR values were obtained by Seahorse technology and plotted over time (*n* = 3 for each condition). All data are shown as mean ± SD.

To explore if exposure of unprimed PMN to EVs of different cellular sources changed the energetic status and oxidative responses, we analyzed the PMN metabolic parameters of oxygen consumption (OCR) and extracellular acidification rates (ECAR) via Seahorse analytics (**Figure 2**). Overall, encounter with EVs neither affected oxidative nor glycolytic responses of bovine PMN, irrespective of the EV source (**Figure 2**).

### 3.3 Exposure of PMN to BUVEC- and *B. besnoiti* tachyzoite-derived EVs induce extracellular DNA release in a NOX-independent manner

**Figure 3:**
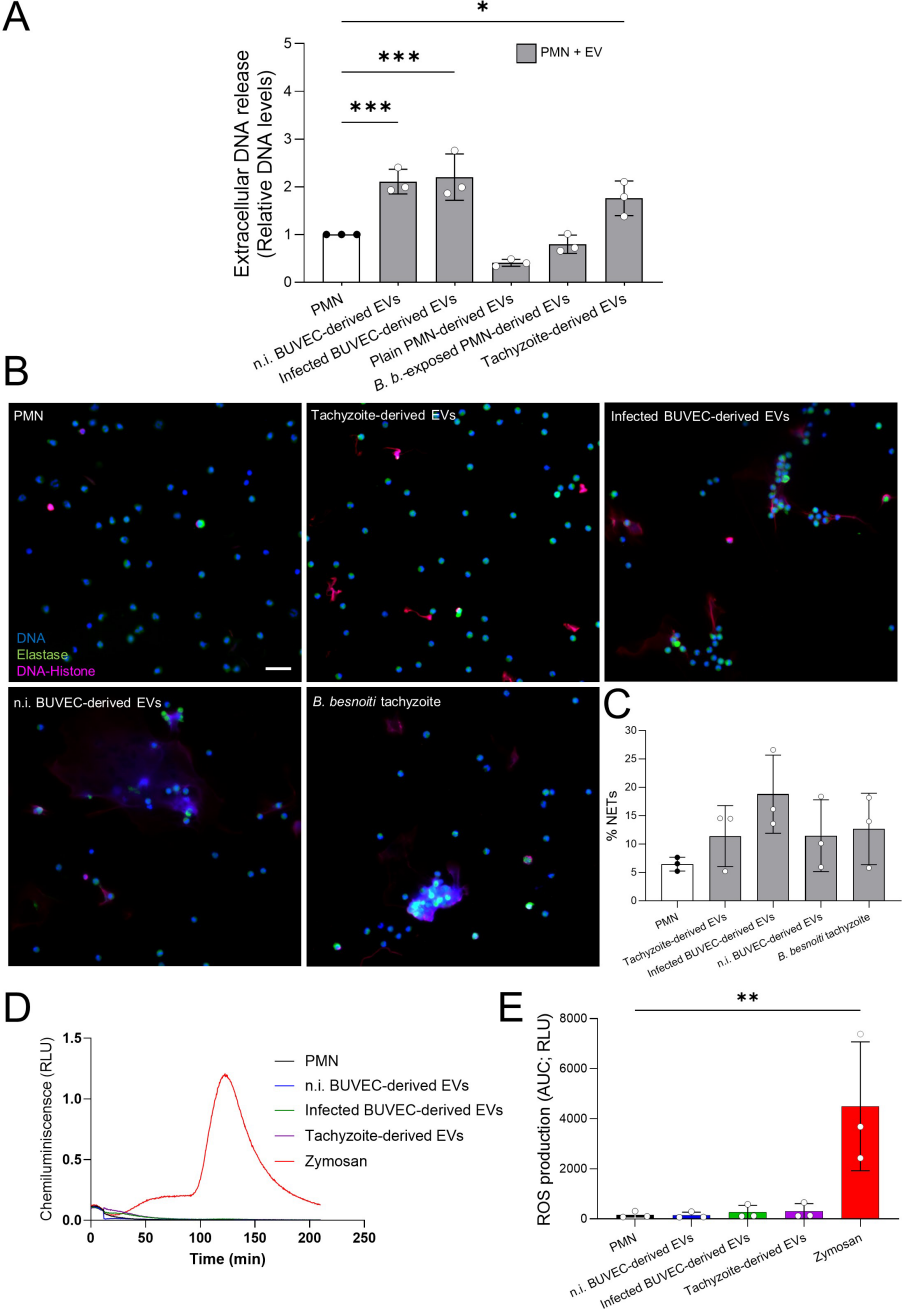
Exposure of bovine PMN to BUVEC- and *B. besnoiti* tachyzoite-derived EVs induced NET formation in a ROS-independent manner. **(A)** Bovine PMN were stimulated with EVs derived from non-infected BUVEC (n.i. BUVEC), *B. besnoiti*-infected BUVEC (Infected BUVEC), unstimulated PMN (Plain PMN), *B. besnoiti* tachyzoite-exposed PMN (*B. b.*-exposed PMN) and from *B. besnoiti* tachyzoites (Tachyzoite) for 4 h After incubation, extracellular DNA was quantified via picogreen-derived fluorescence intensities. All data are shown as mean ± SD; *p*-values were calculated by one-way ANOVA followed by Dunnett´s multiple comparison test. *p < 0.05; **p < 0.01; ***p < 0.001. **(B)** Exemplary immunofluorescence images showing DNA (blue), neutrophil elastase (green) and DNA-histone complexes (magenta) in EVs-exposed PMN. **(C)** The percentage of NET-releasing PMN was calculated via image analysis (Image J, Fiji version); bars represent mean ± SD. **(D, E)**. Representative kinetic and total ROS production of EV-exposed PMN, evaluated by luminol-based assays after 4 h of exposure. Zymosan served as positive control. (*n* = 3). Scale bar = 30 µm.

To address if EV exposure has an impact on PMN effector mechanisms, we first focused on NET formation. Bovine PMN were exposed to EVs derived from non-infected BUVEC, *B. besnoiti*-infected BUVEC, non-stimulated PMN, tachyzoite-exposed PMN and *B. besnoiti* tachyzoites (**Figure 3**). Extracellular DNA quantification based on picogreen-derived fluorescence intensities was performed at 4 h of incubation, thereby rather reflecting a late phase of NET formation. Relative DNA level analysis showed a significant increase of extracellular DNA release only for PMN stimulated with EVs derived from BUVEC (*p* < 0.001) and from *B. besnoiti* tachyzoites (*p* < 0.05) when compared to medium controls (**Figure 3A**). In the former case, EV-driven NET formation revealed independent of the infection status of BUVEC since EVs from non-infected and *B. besnoiti*-infected BUVEC equally induced NET formation. In contrast, PMN-derived EVs failed to induce extracellular DNA release, irrespective of PMN stimulation (**Figure 3A**). Therefore, we focused further experimentation on BUVEC- and *B. besnoiti* tachyzoite-derived EVs. To confirm typical characteristics of NET formation, classical NET markers (NE and histone-DNA) were visualized by immunostaining (**Figure 3B**), applying a semi-automatic image analysis for NET quantification (**Figure 3C**). Here, the presence of extracellular DNA concomitant with histone and NE was confirmed for NET structures from PMN stimulated with BUVEC- and tachyzoite-derived EVs at 4 h (**Figure 3B**). Further analysis revealed a tendency to increase in the percentage of PMN extruding NETs in case of tachyzoite-derived EVs, *B. besnoiti*-infected BUVEC-derived EVs, non-infected BUVEC-derived EVs, and *B. besnoiti* tachyzoite-derived EVs in comparison with control (**Figure 3C**).

To study, if extracellular DNA release coincided with PMN ROS production, total ROS production was measured in PMN stimulated with BUVEC- and tachyzoite-derived EVs (**Figures 3D, E**). However, current data revealed that EVs from all tested sources failed to affect PMN-derived total ROS production (**Figure 3E**). In contrast, stimulation of PMN with zymosan, serving as positive control for ROS synthesis, indeed triggered significant ROS production.

### 3.4 PMN take up EVs from different cellular sources

**Figure 4:**
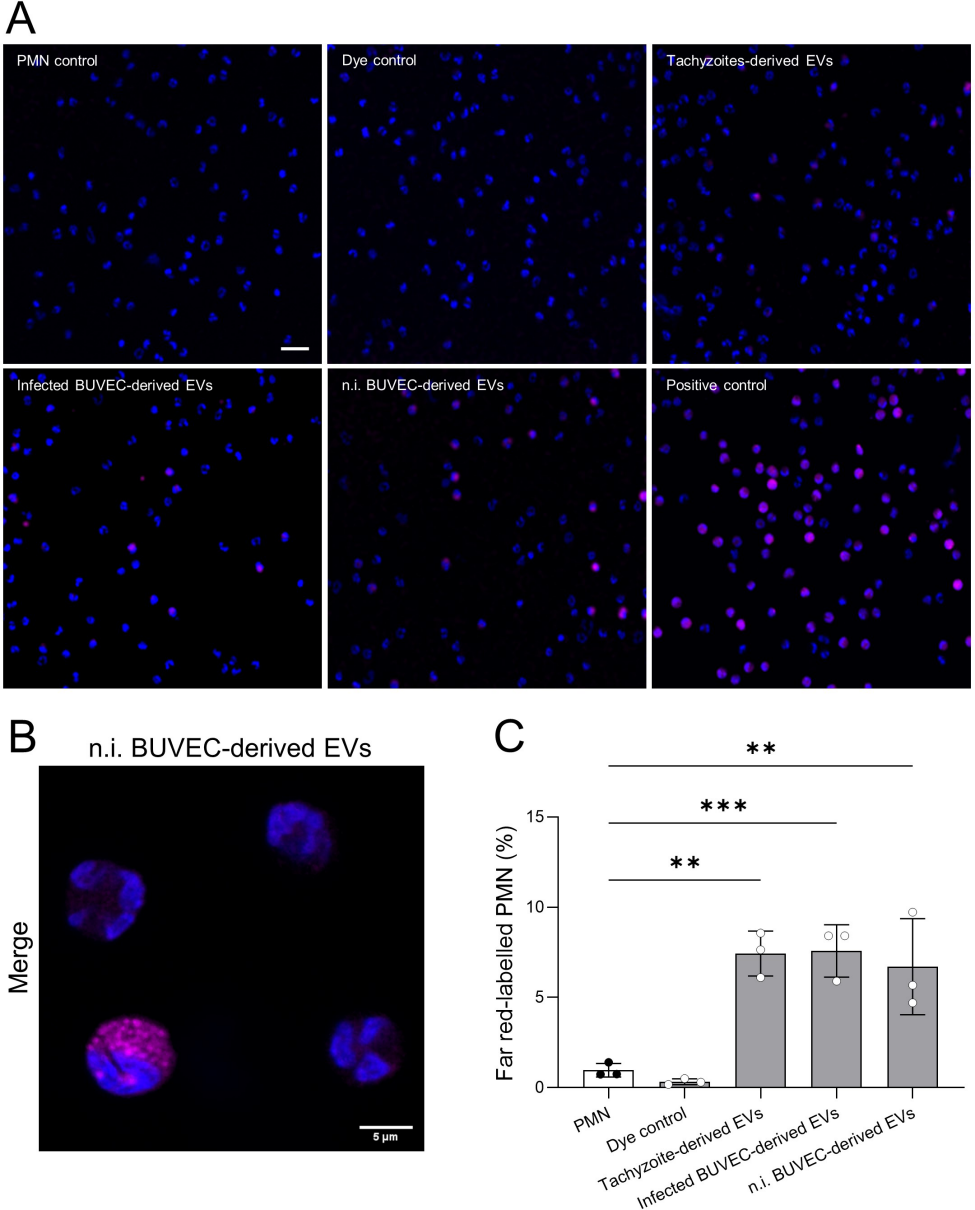
PMN-mediated uptake of far red-labeled EVs. Bovine PMN were exposed to far red-labeled EVs for 6 h, fixed and mounted with fluoromount G (DAPI). **(A, B)** Representative microscopic images depicts PMN (nuclei, blue) with internalized EVs (magenta). **(C)** Semi-automated quantitative analysis of EV internalization showing that PMN equally internalized EVs from all cellular sources. All data are shown as mean ± SD; p-values were calculated by one-way ANOVA followed by Dunnett´s multiple comparison test. **p < 0.01; ***p < 0.001. Scale bar = 20 µm. (*n* = 3).

To study PMN-EV-interactions on the level of EV internalization, bovine PMN (*n* = 3) were co-cultured for 6 h with far red-labeled EVs derived from non-infected BUVEC, *B. besnoiti*-infected BUVEC and *B. besnoiti* tachyzoite (**Figure 4**). PMN-mediated EV uptake was assessed by confocal microscopy (**Figure 4A**) visualizing a rather globular staining within the PMN cytoplasm, most likely reflecting endosomal localization of internalized EVs, as described in the literature (48) (**Figure 4B**). Semi-automated microscopic quantification revealed a significant increase in PMN-derived far red signals upon EV encounter. Thus, almost equal fractions of PMN with far red signals were detected in case of EVs from tachyzoites, *B. besnoiti*-infected BUVEC and non-infected BUVEC (**Figure 4C**).

### 3.5 EV exposure to PMN selectively induces the release of IL-1β and IL-6 but not of CXCL8

**Figure 5:**
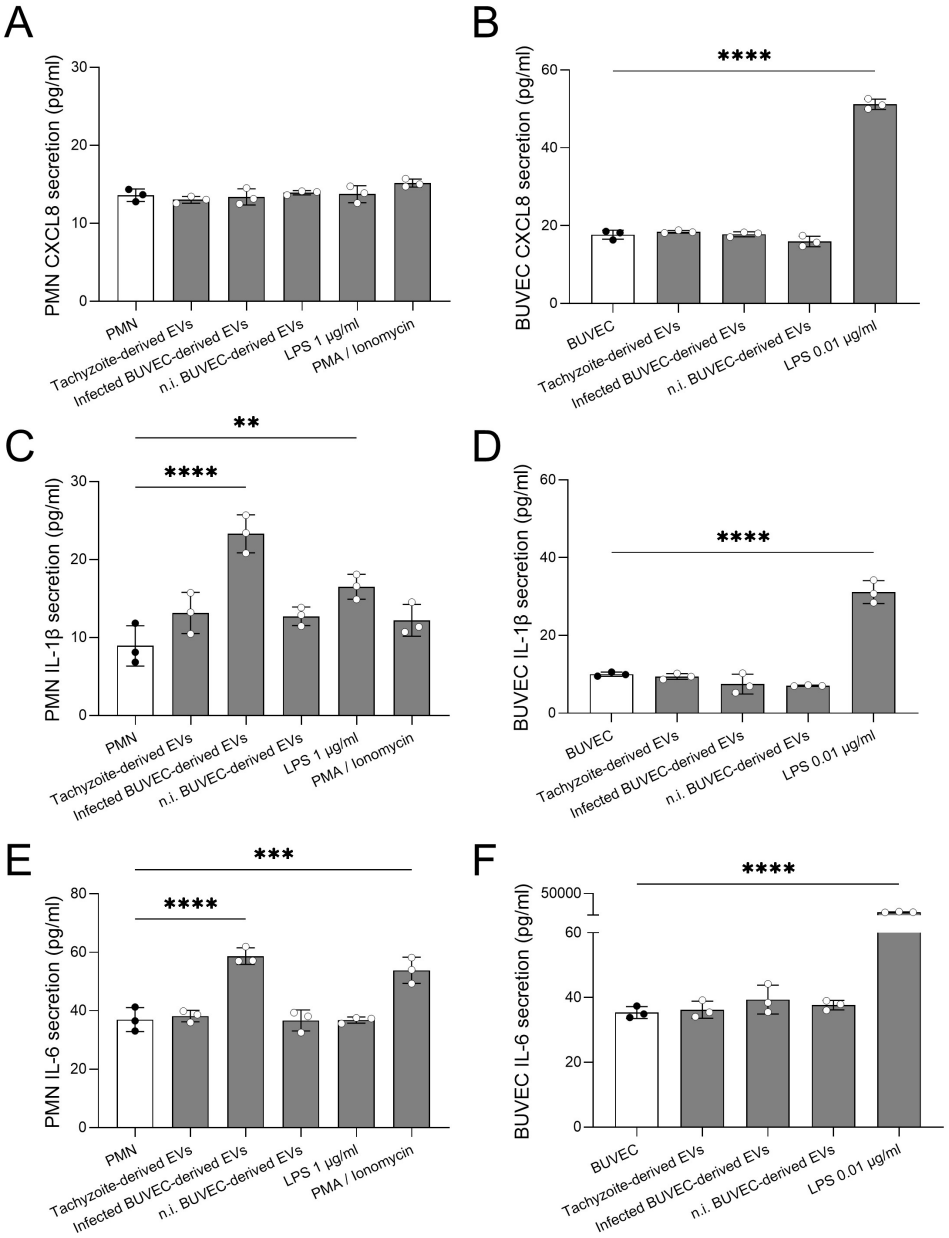
Selected EV exposure induced IL-1β and IL-6 release in bovine PMN. Bovine PMN or BUVEC were exposed to EVs from different cellular sources for 4 h (PMN) and 24 h (BUVEC). Thereafter, CXCL8 **(A, B)**, IL-1β **(C, D)**, and IL-6 **(E, F)** was quantified via commercially available ELISAs in co-culture-derived supernatants. Stimulation with LPS and PMA/ionomycin was used for positive controls. All data are shown as mean ± SD; p-values were calculated by one-way ANOVA followed by Dunnett´s multiple comparison test. (n = 3). **p < 0.01; *** *p* < 0.001; **** *p* < 0.0001.

Since EVs are well-documented for their role in intercellular communication, we explored their capacity to induce inflammatory responses in PMN and BUVEC by assessing the release of IL-1β, IL-6 and CXCL8. These inflammatory mediators were quantified via commercially available ELISAs in supernatants from both PMN and BUVEC being exposed to EVs from BUVEC and *B. besnoiti* tachyzoites (**Figure 5**). At 4 and 24 h of exposure for PMN and BUVEC, respectively, only trace amounts of IL-1β, IL-6 and CXCL8 were detected in supernatants of PMN (**Figures 5A, C, D**) and BUVEC (**Figures 5B, D, F**). Nevertheless, PMN-derived IL-1β and IL-6 release was significantly increased after PMN exposure to EVs derived from *B. besnoiti*-infected BUVEC (*p* = <0.0001 and *p* = <0.0001, respectively, **Figures 5C, E**). In contrast, EVs failed to induce CXCL8 secretion in PMN. BUVEC stimulation with EVs of different origin all failed to affect endothelial IL-1β, IL-6 and CXCL8 release. Interestingly, PMN showed differential cytokine secretion depending on the stimuli initially used for positive controls. Thus, LPS induced an increase in IL-1β secretion while stimulation with PMA/ionomycin enhanced IL-6 release. Furthermore, LPS worked as a positive stimulus for BUVEC by inducing an enhanced secretion of both IL-1β and IL-6.

## 4 Discussion

NET formation is an effector mechanism of PMN acting against invasive pathogens. We recently demonstrated that *B. besnoiti* tachyzoites effectively induce PMN activation and NET formation in the bovine system thereby showing that this parasite-driven process depends on classical parameters like metabolic responses (31), MPO/NE activity and ROS production (30), in addition to P_2_X_1_ purinergic- (28), AMPK- and autophagy-related signaling (49, 50). In the present study, we investigated the role of EVs from differential cellular origin in bovine PMN activation and effector functions thereby considering *i)* the PMN energetic state, *ii)* NET formation, *iii)* EV internalization and *iv)* cytokine/chemokine secretion. Given that *B. besnoiti* primarily infects endothelial cells *in vivo*, we analyzed effects of EVs derived from infected and non-infected bovine endothelial cells, from tachyzoite-stimulated and unstimulated PMN and from tachyzoite stages. By intention, we here aimed to study immediate and unprimed reactions.

Encounter of PMN with *B. besnoiti* stages drives NET formation (28, 30–32, 49, 50), however, the specific triggers of *B. besnoiti*-induced NET formation are still unknown. In that context, EVs have come into interest based on their ability to mediate communication between parasites and cells (51). EVs contain proteins, RNA/DNA, lipids and metabolites and EV-derived molecules were shown to be involved in drug resistance, cell growth regulation and immune cell modulation (52). EVs are of a complex nature, therefore a plethora of protocols and guidelines on their isolation and characterization exist using differential centrifugation/ultracentrifugation, affinity-based capture (such as antibody-coated magnetic beads or resins), ultrafiltration, size-exclusion chromatography and Nano-Flow cytometry, among others (53, 54). In the current work, we used differential centrifugation at low-speed to eliminate high molecular contaminants, ultrafiltration to eliminate proteins and to enrich EVs and size-exclusion chromatography to purify and recover EVs. The latter process was performed with the help of an automated collector (IZON), thereby achieving an improved reproducibility, speed and simplicity of EV isolation. In the current study, EV characterization was performed following MISEV 2018 guidelines (54), considering parameters like EV size, concentration, membrane protein biomarkers and morphology. Overall, particles from all used cellular sources showed a mean size of 70 nm, thereby fitting well to size ranges described for small EVs in literature (30-150 nm; 41, 47). For further characterization, all EV samples were tested for the presence of the tetraspanins CD9 and CD81 as EV markers. Western blot analyses proved BUVEC- and PMN-derived EVs as positive for both CD9 and CD81 proteins. Moreover, the expected size and morphology of EVs from selected sources were verified by TEM, thereby revealing for the first time *B. besnoiti* tachyzoite-derived EVs. Taken together, these results confirmed that the particles isolated from BUVEC-, PMN- and *B. besnoiti* tachyzoite-derived supernatants were indeed EVs in terms of size, morphology, and protein components.

Host-parasite communication via EVs has extensively been analyzed in the last decade (38, 40, 41, 52, 55–58). In general, pathogen encounter seems to foster EV release by effector cells. Thus, infections with *Plasmodium* stimulated EV release from endothelial cells, platelets, and red blood cells (RBCs). In agreement, exposure of PMN to *B. besnoiti* tachyzoites led to a rise in PMN EV secretion. Interestingly, enhanced EV levels correlated with severe illness both in rodent malaria model and in malaria patients (59, 60). EVs originating from parasite-infected RBCs activated the innate immune response via pro- and anti-inflammatory cytokines in *P. falciparum and P. berghei* infections. These EVs may also play a role in vascular activation and dysfunction, thereby facilitating parasite sequestration and associated pathology (59, 60). Moreover, *Cryptosporidium parvum* infection of human cells lines (H69 and 603B cells) induced an increment of luminal EV release from biliary and intestinal epithelium. These EVs carried antimicrobial peptides from epithelial cell origin (such as beta-defensin 2), helping to decrease sporozoite viability and infectivity both *in vitro* and *ex vivo* (61). In general, the extent of EV production and/or nature of content may vary depending on the cell type and activation status. In line with current results denying any infection-driven increase of endothelial EV release, treatments of HUVEC with TNF-α did not affect the production, size or morphology of EVs (62). In a another study, unstimulated human PMN secreted lower EV quantities than PMN exposed to different classes of physiological stimuli, such as fMLP, LPS and TNF-α (63). Given that GM-CSF and IFNγ failed to induce EV release, stimuli-specific reactions were suggested (63).

After having confirmed EV characteristics, EV samples from BUVEC, PMN and *B. besnoiti* tachyzoites were studied for their effects on glycolytic and oxidative responses, NET formation, ROS production and chemokine/cytokine secretion in unstimulated PMN. Unexpectedly, current findings revealed that EVs from all cellular sources failed to affect PMN metabolic (glycolytic and oxidative) responses and ROS production. Of note, we aimed to characterize immediate reactions of resting bovine PMN and therefore worked with non-activated cells. In line, EVs from both unstimulated human PMN and opsonized particle-activated PMN failed to affect ROS production in resting PMN (64, 65). By contrast, EV treatments decreased ROS production in PMA-pre-activated PMN (64). In contrast to current data, EVs derived from human PMN stimulated with another protozoa (*Entamoeba histolytica)* triggered a significant increase of PMN ROS production (65). However, when PMN were pre-stimulated with PMA or *E. histolytica* trophozoites and then exposed to EVs from unstimulated or *E. histolytica*-stimulated PMN, a significant decrease or no change in ROS production was observed, respectively (65). These data clearly indicate that several factors like the pre-stimulus status of donor or receiver PMN and the type of stimulus highly matter in EV-mediated PMN reactions. Current data showed that resting bovine PMN failed to respond to EVs of different cell sources on the level of ROS or metabolic changes. In addition, stimulation of PMN with *B. besnoiti-*exposed PMN*-*derived EVs or unstimulated PMN-derived EVs also failed to significantly drive extracellular DNA release. Nevertheless, in case of NET formation, EV exposure of resting PMN showed a differential reaction pattern compared to ROS. Here, BUVEC- and tachyzoite-derived EVs indeed fostered NET release. The fact, that PMN-derived EVs failed to drive NET formation in the current experimental setting is in line with former data on another protozoan parasite, stating that unstimulated PMN-derived EVs and EVs derived from *E. histolytica*-stimulated PMN did not induce NET formation in resting PMN (65), thereby highlighting again the importance of the priming state for EV-exposed PMN. Of note, endothelial cells are well-known as effective producers of EVs, thereby communicating with all kinds of cells (37, 39, 62). In the current study, BUVEC-derived EVs triggered NET formation in resting bovine PMN, independent of the infection status of BUVEC, but being accompanied by a lack of ROS production, thereby indicating NOX-independent NET formation. NOX-independent NET formation was recently described to be triggered by an increase in calcium and mitochondrial ROS, activating PAD4 and histone citrullination, concomitant with ERK1/2 and JNK pathway activation (16). Interestingly, EVs were described to carry miRNAs and other signaling molecules, which are able to activate the JNK and ERK1/2 signaling pathway (66–68). Furthermore, EVs may also transport trace amounts of ROS from their progenitor cell (65). However, the potential mechanism being involved in NOX-independent NET formation triggered by BUVEC- and *B. besnoiti* tachyzoite-derived EVs awaits further investigation.

EVs participate in immune signaling due to their capacity to transport both pro-inflammatory and anti-inflammatory cytokines to designated target cells, in addition to their ability to induce the secretion of these cytokines from recipient cells (69). Current data showed that bovine PMN increased IL-1β and IL-6 secretion in a stimulus-dependent manner since exclusively EVs from *B. besnoiti*-infected BUVEC fostered the release of these cytokines. Meanwhile, BUVEC failed to react by IL-1β, IL-6 or CXCL8 release after exposure to EVs, independent of the cellular source. Regarding PMN, this finding correlates with data on other innate immune cells like macrophages, monocytes or dendritic cells. Hence, *T. gondii*-derived EVs were shown to drive resting macrophage activation by increasing IL-12, TNFα and INFγ secretion (70). Moreover, *Leishmania donovani* promastigote-derived EVs modulated the cytokine response of monocytes by enhancing IL-10 expression but suppressing TNFα synthesis, while EV-exposed monocyte-derived dendritic cells (DCs) showed diminished levels of IL-10, IL-12p70, TNFα (71). Moreover, *in vivo* administration of EVs from *T. gondii* antigen-stimulated DCs led to an increase in Th1 cytokines (including IL-2 and IFN-γ) with concurrent diminishment of Th2 cytokines (e. g. IL-4, IL-5, and IL-10) (72). This literature indicated that IL-1β and IL-6 are mediating host protection against parasites infection, activating and inflammatory responses. For instance, IL-6 deficient mice were found more susceptible to *T. gondii* infection, allows increased parasite growth (73). Moreover, *T. gondii* is able to suppress IL-1β production from human PMN as an evasion mechanism of host defense (74). It is important to highlight that this EV-cytokine-communication is bidirectional. Hence, PMN EV production can also be induced by CXCL8 and TNF-α (75). Of note, several host molecules and proinflamatory cytokines induces or boost NET formation (76, 77). Both macrophage-derived and plasmacytoid dendritic cells (pDCs)-derived type I IFNs promote NET release (77). Additionally, proinflammatory cytokines such as tumor necrosis factor (TNF), IL-1β, and IL-12, which are secreted by leukocytes during inflammation, have been shown to enhance NET formation (77). Furthermore, patients with systemic inflammatory response syndrome possess higher plasma levels of IL-8, IL-1β, and TNF-α, which induce NET formation in PMN from healthy individuals (78). These findings underscore the critical role of cytokines in modulating NET formation, particularly in inflammatory conditions.

To fulfill their function in cell-to-cell communication, EVs interact with target cells through receptor-ligand binding mechanisms or by internalization via different endocytic mechanisms, which include clathrin-dependent endocytosis and clathrin-independent routes like caveolin-mediated uptake, macropinocytosis, phagocytosis, and lipid raft-mediated internalization (48). Therefore, we tested if EVs from the different cellular sources are taken up by resting bovine PMN. Indeed, confocal microscopy confirmed that BUVEC- and *B. besnoiti*-derived far red-labeled EVs were internalized by PMN leading to cytoplasmic localization in exposed PMN. As expected, PMN-derived EV uptake occurred irrespective of the EV source. In principle, these data match with findings on human PMN or other innate immune cell types. Thus, *E. histolytica*-derived EVs fused with the PMN cell membranes and were internalized into the cytoplasm by human PMN (65). Moreover, cytoplasmic internalization of immature DC-derived EVs was described for unstimulated DCs (46) and *T. gondii*-derived EVs were taken up into the cytoplasm of RAW264.7 macrophages (70).

To summarize, we here showed that bovine PMN enhanced their EV production when being confronted to *B. besnoiti* stages. Bovine PMN showed no ROS production or glycolytic/oxidative responses when being exposed to EVs from differential cellular origin. Importantly, NET formation and IL-1β/IL-6 secretion were upregulated by *B. besnoiti* infected-endothelium- and *B. besnoiti* tachyzoite-derived EVs.

## Data Availability

The raw data supporting the conclusions of this article will be made available by the authors, without undue reservation.
